# Plasma Amino Acid Profiles and Clinical Outcome in Patients with Traumatic Brain Injury: A Study Protocol

**DOI:** 10.31661/gmj.v13i.2944

**Published:** 2024-02-23

**Authors:** Alireza Gheflati, Mostafa Shahraki Jazinaki, Mahlagha Nikbaf-Shandiz, Pegah Rahbarinejad, Hamid Rezaee, Saeid Eslami, Majid Khadem-Rezaian, Alireza Sedaghat, Mohsen Nematy, Mahdi Shadnoush, Ali Jafarzadeh Esfehani, Fatemeh Keyfi, Zachary S. Clayton, Abdolreza Norouzy

**Affiliations:** ^1^ Department of Nutrition, Faculty of Medicine, Mashhad University of Medical Sciences, Mashhad, Iran; ^2^ Student Research Committee, Tabriz University of Medical Sciences, Tabriz, Iran; ^3^ Department of Neurosurgery, Faculty of Medicine, Mashhad University of Medical Sciences, Mashhad, Iran; ^4^ Department of Medical Informatics, Faculty of Medicine, Mashhad University of Medical Sciences, Mashhad, Iran; ^5^ Pharmaceutical Research Center, Mashhad University of Medical Sciences, Mashhad, Iran; ^6^ Department of Community Medicine, School of Medicine, Mashhad University of Medical Sciences, Mashhad, Iran; ^7^ Department of Anesthesiology, Mashhad University of Medical Sciences, Mashhad, Iran; ^8^ Metabolic Syndrome Research Center, Mashhad University of Medical Sciences, Mashhad, Iran; ^9^ Department of Clinical Nutrition, Faculty of Nutrition and Food Technology, Shahid Beheshti University of Medical Sciences, Tehran, Iran; ^10^ Department of Medical Laboratory Sciences, Varastegan Institute for Medical Sciences, Mashhad, Iran; ^11^ Division of Metabolic Disorder, Pardis Clinical and Genetic Laboratory, Mashhad, Iran; ^12^ Department of Integrative Physiology, University of Colorado, Boulder, CO, USA

**Keywords:** Traumatic Brain Injury, Mortality, Amino Acid, Clinical Protocols

## Abstract

Background: The most common cause of cognitive and behavioral impairments, disability, and mortality around the world is traumatic brain injury (TBI). The imbalance between cerebral metabolism and inflammation leads to protein breakdown and induces altered concentrations of serum amino acids, which can serve as a diagnostic and prognostic sign in patients with TBI. This study aimed to examine the alterations in plasma amino acid concentrations and their relation to clinical outcomes in patients with TBIs.Materials and Methods: At completion, this study will assess 107 patients suffering from TBI aged 18 to 65. Plasma amino acid concentrations, anthropometric indices, and clinical outcome parameters including Acute Physiology and Chronic Health Evaluation (APACHE) II, Sequential Organ Failure Assessment (SOFA), Nutrition Risk in the Critically ill (Nutric) score, Glasgow coma scale (GCS), Intensive Care Unit (ICU) discharge time, mechanical ventilator duration, and mortality rate will be assessed at the beginning of the study, day 7, and day 14.Conclusion: This longitudinal study will provide evidence for further clinical trials and observational studies on amino acid supplementation and TBI. The results of this study could inform future treatment strategies for TBI patients.

## Introduction

Traumatic brain injury (TBI) is the leading cause of cognitive and behavioral impairments, disability, and mortality around the world [1]. As reported by the Global Burden of Diseases (GBD), TBI led to 8.1 million (95% UI 6·0-10·4 million) years of life lost (YLLs) in 2016 [2]. TBI can contribute to a variety of pathophysiological states [3]. For example, TBI can lead to blood-brain barrier rupture, neuroinflammation, neurodegeneration, and ischemic injury, which induce significant changes in cerebral metabolism and exacerbate systemic and neurological inflammation [4].

The concomitant imbalance of cerebral metabolism and inflammation results in protein breakdown, which alters plasma amino acid concentrations [5]. Therefore, measuring amino acid concentrations in patients with TBI is valuable for diagnostic and prognostic purposes [6].

Previous studies have shown that brain tissue after TBI contained up to 50-fold more glutamate and aspartate [7]. Serine, taurine, and alanine levels in the serum also decreased significantly 24 hours after TBI but returned to pre-injury levels after four days [8]. TBI reduces serum glycine levels and disturbs redox balance, ultimately leading to neuronal death [8]. Patients with TBI also had lower levels of branched-chain amino acids (BCAAs) including leucine, isoleucine, and valine relative to healthy individuals, likely as a result of the metabolic cascade that developed after the injury [9].

Alternatively, Intensive care unit (ICU) length of stay (LOS) and ventilator dependency are associated with plasma taurine concentration [10]. Furthermore, low taurine levels were associated with high blood lactate, which may indicate a state of heightened metabolic demand [10]. A better understanding of changes in amino acid profiles among patients with TBI will help clinicians more accurately estimate amino acid requirements and provide optimal nutritional support [11].

To the best of our knowledge, prior studies have only examined the alterations of select amino acids after TBI. No studies have comprehensively assessed plasma amino acid concentrations in patients with TBI.

This study will be the first to comprehensively assess plasma amino acid levels in patients with TBI and relate these values to clinical outcomes including Glasgow coma scale (GCS), mechanical ventilator duration, ICU discharge time, and mortality rate. The results of this study will inform whether particular amino acid profiles are useful biomarkers for TBI.

## Materials and Methods

**Figure-1:**
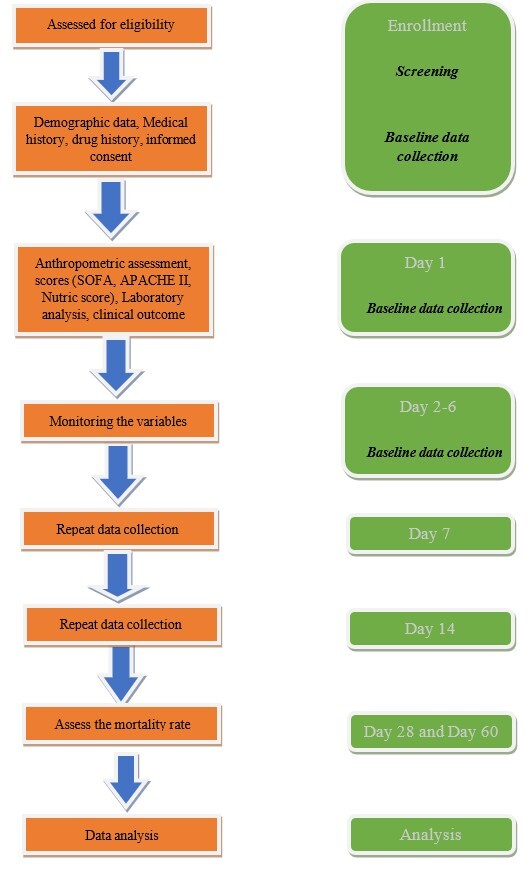
Figure 1. Study procedure flow sheet

**Table:** Table1. Timeline and Applied Tests

Time Point				Days		
		0	7	14	28	60
Enrolment						
Eligibility screening		*				
Informed consent		*				
Medical interview		*				
Blood collecting		*	*	*		
Assessment						
Demographic		*				
Laboratory assessment (20 types of plasma amino acid)		*	*	*		
Clinical outcome: GCS, vital signs, electrolyte balance						
Anthropometric measurements	Weight	*	*	*		
	Height	*	*	*		
	BMI	*	*	*		
	Fat mass	*	*	*		
	Lean body mass	*	*	*		
	MAC	*	*	*		
SOFA		*	*	*		
APACHE II		*	*	*		
Nutric score		*		*		
GOS		*		*		
Mortality status				*	*	*
Days of hospital stay				*	*	*
Dietary intake						
Need mechanical ventilator		*	*	*		

**BMI:** Body mass index; **GCS:** Glasgow coma scale; **SOFA:** Sequential Organ Failure Assessment; **APACHE II:** Acute Physiology and Chronic Health Evaluation II; **Nutric score:** Nutrition Risk in the Critically Ill score; **MAC:** Mid-arm circumference

After the completion of this study, 107 individuals with TBI hospitalized in the ICU will be recruited. Participants will be selected according to inclusion and exclusion criteria by a qualified neurosurgeon from adult trauma wards referred to Shahid Kamyab, Taleghani, and Shahid Hasheminejad hospitals in Mashhad, Iran.

This project was initiated in September 2022 and will likely be completed in July 2023. The details of this study protocol are depicted in the flow diagram (Figure-1).

1. Study Design and Setting

Upon completion of this study, a total of 107 patients who have been diagnosed with subarachnoid hemorrhage (SAH), subdural hemorrhage (SDH), epidural hemorrhage (EDH), brain edema, intracerebral hemorrhage (ICH), or intraventricular hemorrhage (IVH) will be recruited by the neurosurgeon. According to inclusion and exclusion criteria, patients will be chosen.

2. Eligibility Criteria

The inclusion criteria for this study will be a) patients with TBI aged 18 to 65 years; b) being admitted to a neurocritical care unit with any type of TBI diagnosis; c) moderate to severe TBI (7≤GCS≤12), and e) signing the informed consent form by the patient or family members.

The exclusion criteria will be: a) severe and active bleeding; b) taking inotropic and corticosteroid medications; c) history of any kind of autoimmune, cancer, and metabolic diseases; d) refusal to continue the study by the patient, parents, or relatives members; e) acute or chronic liver failure; f) pregnancy and breastfeeding; g) chronic renal failure, and h) acute renal failure requiring dialysis.

3. Ethics Approval

The Medical Ethics Committee of the Mashhad University of Medical Science approved this longitudinal study protocol. (IR.MUMS.MEDICAL.REC.1400.589).

4. Study Assessment

4.1. Clinical Outcome Assessment

Clinical measurement tools including GCS, Sequential Organ Failure Assessment (SOFA), Acute Physiology and Chronic Health Evaluation (APACHE) II score, and Nutrition Risk in the Critically ill (Nutric) score, ICU discharge time, and mechanical ventilator duration will be recorded by a nutritionist at baseline, middle (7th day) and end of the study (14th day). Moreover, 28-day and 60-day in-hospital and out-of-hospital mortality will be recorded during the study. Demographic data, social and medical background (current employment, place of residence, marital status) will be collected by a neurosurgeon.

4.2. Anthropometric Assessment

Anthropometric parameters will be measured at baseline, 7th day, and 14th day by a well-trained nutritionist. A bed scale (Balas Company) will be used to measure body weight, also the ulnar length will be used to estimate height. The mid-arm circumference will be measured at the mid-point between the elbow and apex of the shoulder by a non-stretched tape. Fat and fat-free masses will be measured by bioelectrical impedance analysis (s10, in Body Company, South Korea). The body mass index will be calculated based on the ratio of the weight in kilograms to the square of the height in meters. All anthropometric indices will be collected by a trained nutritionist. The study timeline is demonstrated in Table-1.

4.3. Laboratory Assessment

Five milliliters (5 cc) of venous blood samples will be taken in EDTA-containing tubes at baseline, 7th day, and 14th day, at ~7:00 am, for assessment of plasma amino acids. Blood samples will be centrifuged immediately to separate the plasma sample from the whole blood. The plasma will be stored at -20 °C after being divided into aliquots and placed in microtubes. The samples will then be placed in a freezer at -80 °C to be ana­lyzed for the concentration of 18 types of plasma amino acids (glycine, glutamic acid, valine, asparagine, lysine, serine, glutamine, isoleucine, threonine, ar­ginine, alanine, tyrosine, methionine, trypto­phan, phenylalanine, aspartic acid, leucine, and histidine) and two metabolites of arginine (ornithine and citrulline). After precolumn derivatization with o-phthalaldehyde (OPA), the concentrations of plasma amino acids will be measured using reverse-phase high-performance liquid chromatography (RP-HPLC) on a C18 column with a fluorescence detector. Briefly, 100 µL of plasma will be mixed with 200 µL of the internal standard. The tubes will be centrifuged for 5 minutes at 12000 rpm. Twenty µL of supernatant will be added to 100 µL buffer solution before the addition of 20 µL of OPA reagent. Following, samples will be blended with 200 µL of distilled water, and 20 µL of the mixture will be injected into the HPLC system (Agilent Technologies 1260 infinity series, USA) for analysis.

5. Power Calculation and Sample Size Estimates

The sample size was computed based on Oudemans-van Straaten et al. (2010) [12] based on 5% Type I error (α=0.05), 0.093 as effect size (d=0.093) (96 persons). Finally, the sample size was increased to 107 patients to account for 10% attrition.

6. Statistical Methods

To assess the normality of quantitative variables, the Kolmogorov-Smirnov test will be performed. For normally distributed data, the quantitative data will be presented as mean ± standard deviation (SD), and for variables with non-normal distribution, including median and interquartile range (IQR). The qualitative data will be demonstrated in the form of frequency and percentage. Quantitative variables will be compared between the survived and deceased groups using the student t-test or the Mann-Whitney test. The chi-square or Fisher’s exact test will be used to analyze the comparison of qualitative variables between the surviving and deceased patients. The relation among quantitative variables will be investigated by Pearson or Spearman correlation coefficient. The Kaplan-Meier test will be conducted to compare the survival rate between amino acid-sufficient and deficient patients based on the log-rank test of survival time. The test results will be considered statistically significant when the P-value is less than 0.05. The data will be analyzed using the SPSS Software Version 23 (IBM Inc, Chicago, IL, USA).

## Discussion

Until now, the relation of select amino acids with mortality and several clinical outcomes have been discussed separately, however, there is little information available regarding the prevalence of all essential and non-essential amino acid deficiencies and the relation between amino acid profiles and main clinical parameters among patients with TBI.

Physical damage to brain tissue caused by TBI could result in brain dysfunction [13] including ischemia, glutamate excitotoxicity, changes in neurotransmitter function, neuroinflammation, altered brain metabolism, a heightened pro-inflammatory state, and protein breakdown [14]. Previous studies have indicated protein catabolism/anabolism imbalance in patients with TBI [15][16], and have identified serum amino acid concentrations as the main predictor of mortality in patients with acute TBI. Recently, serum amino acid levels were shown to be associated with brain tissue concentrations, and as such, serum amino acids may be prognostic indicators of TBI [6]. For example, increased glutamate receptor activation and redox imbalance are observed in patients with TBI, which can lead to cell death within the brain and ultimately neurological dysfunction [17][18].

Studies indicated that the glutamate toxicity side effects can be reversed by elevated levels of glycine, taurine, and serine in brain tissue through their role in mitochondrial function regulation [18][19]. Moreover, glycine levels in brain tissue and serum glycine concentrations in patients with TBI may be linked to the pathology of oxidative stress caused by TBI, as it is a small neutral amino acid that could easily diffuse through a disrupted blood-brain barrier [20]. Alternatively, increased glycine levels in brain tissue and decreased serum glycine levels after TBI may be due to the antioxidant property of this amino acid. In addition, glycine and serine interact with glutamate receptors [21]. Moreover, glycine and cytosine are precursors of glutathione synthesis, a primary antioxidant system, and increasing levels of these amino acids may be a means to reduce TBI-mediated oxidative stress [22]. The critical amino acids serine, glycine, and alanine produce glucagon, which is essential in TBI-stimulated glycogen metabolism [23]. Some studies have observed reduced proline levels in patients with TBI [24], suggesting that proline levels may be linked to TBI pathology, whereas other investigations have shown anti-inflammatory effects of proline in the cerebral cortex [25]. An experimental study reported low serum citrulline levels in a model of severe TBI in rats, but the exact mechanism mediating this response was unclear [21]. On the other hand, there is evidence that L-citrulline has a protective effect on cerebral arteries [26]. The concentration of circulating amino acids is likely to be considered a potential prognostic marker for patients with TBI, as there is a negative nitrogen balance in TBI patients. The importance of the relation between plasma amino acid levels and mortality and other paraclinical biomarkers has been mentioned in previous investigations. Past studies showed a possible association between the higher concentrations of some amino acids like homocysteine and magnetic resonance imaging (MRI) finding as silent brain infracts, atrophy of the brain, white matter hyperintensity, elevated risk of subclinical stroke, and other neuropsychiatric diseases [27][28][29][30]. On the other hand, according to Rahmani et al. a rise in homocysteine levels was significantly associated with an increase in the rate of mortality and a significant decrease in GCS [31]. Also, based on the study of Vuille-Dit-Bille et al. an increase in intracranial pressure (ICP) following TBI is associated with a rise in leucine and isoleucine levels [32]. In addition, low plasma taurine is correlated with a longer mechanical ventilation dependency and length of ICU stay [10]. Therefore, it can be expected that amino acid levels can be a good predictor of mortality and other paraclinical data.

## Acknowledgements

The Mashhad University of Medical Sciences will provide financial support for this study.

## Conflict of Interest

No conflicts of interest were declared by the authors.
